# Supplementary material for: Relationship of Daily Coffee Intake with Vascular Function in Patients with Hypertension
Source: Nutrients. 2022 Jun 29;14(13):2719. doi: 10.3390/nu14132719 (PMC9268420; doi:10.3390/nu14132719)
Supplement: Supplementary file 1 [file nutrients-14-02719-s001.zip › nutrients-1764771-supplementary.pdf]

nutrients

MDPI

Relationship of Daily Coffee Intake with Vascular Function in Patients with Hypertension

Brief title: Coffee and Vascular Function in Hypertension

Takayuki Yamaji, MD, PhD;[1] Takahiro Harada, MD, PhD;[1] Yu Hashimoto, MD;[1] Yukiko Nakano, MD, PhD;[1] Masato Kajikawa, MD, PhD;[2] Kenichi Yoshimura, PhD;[2,3] Chikara Goto, PhD;[4] Aya Mizobuchi, MS;[5] Shunsuke Tanigawa, MS;[5] Farina Mohamad Yusoff, MD, PhD;[5] Shinji Kishimoto, MD, PhD;[5] Tatsuya Maruhashi, MD, PhD;[5] Ayumu Nakashima, MD, PhD;[6] Yukihito Higashi, MD, PhD, FAHA[2,5]

[1]Department of Cardiovascular Medicine, Graduate School of Biomedical Sciences, Hiroshima University, Hiroshima, Japan
[2]Division of Regeneration and Medicine, Medical Center for Translational and Clinical Research, Hiroshima University Hospital, Hiroshima, Japan
[3]Department of Biostatistics, Medical Center for Translational and Clinical Research, Hiroshima University Hospital, Hiroshima, Japan
[4]Department of Rehabilitation, Faculty of General Rehabilitation, Hiroshima International University, Hiroshima, Japan
[5]Department of Regenerative Medicine, Research Institute for Radiation Biology and Medicine, Hiroshima University, Hiroshima, Japan
[6]Department of Stem Cell Biology and Medicine, Graduate School of Biomedical Sciences, Hiroshima University, Hiroshima, Japan

Address for correspondence: Yukihito Higashi, MD, PhD, FAHA
Department of Regenerative Medicine,
Research Institute for Radiation Biology and Medicine, Hiroshima University
1-2-3 Kasumi, Minami-ku, Hiroshima 734-8551, Japan
Phone: +81-82-257-5831 Fax: +81-82-257-5831
E-mail: yhigashi@hiroshima-u.ac.jp

Supplemental Methods

Measurement of FMD and NID

A blood pressure cuff was placed around the forearm of each subject. The brachial artery was scanned longitudinally 5 to 10 cm above the elbow. When the clearest B-mode image of the anterior and posterior intimal interfaces between the lumen and vessel wall was obtained, the transducer was held at the same point throughout the scan by using a special probe holder (UNEX Co.) to ensure consistency of the imaging. Depth and gain setting were set to optimize the images of the arterial lumen wall interface. When the tracking gate was placed on the intima, the artery diameter was automatically tracked, and the waveform of diameter changes over the cardiac cycle was displayed in real time using the FMD mode of the tracking system. This allowed the ultrasound images to be optimized at the start of the scan and the transducer position to be adjusted immediately for optimal tracking performance throughout the scan. Pulsed Doppler flow was assessed at baseline and during peak hyperemic flow, which was confirmed to occur within 15 seconds after cuff deflation. Blood flow velocity was calculated from the color Doppler data and was displayed as a waveform in real time. Baseline longitudinal images of the artery were acquired for 30 seconds, and then the blood pressure cuff was inflated to 50 mm Hg above systolic pressure for 5 minutes. The longitudinal image of the artery was recorded continuously until 5 min after cuff deflation. Pulsed Doppler velocity signals were obtained for 20 sec at baseline and for 10 sec immediately after cuff deflation. Changes in brachial artery diameter were immediately expressed as percentage change relative to the vessel diameter before cuff inflation. FMD was automatically calculated as the percentage change in peak vessel diameter from the baseline value. Percentage of FMD [(Peak diameter - Baseline diameter)/Baseline diameter] was used for analysis. Blood flow volume was calculated by multiplying the Doppler flow velocity (corrected for the angle) by heart rate and vessel cross-sectional area (-r^2). Reactive hyperemia was calculated as the maximum percentage increase in flow after cuff deflation compared with baseline flow.

The response to nitroglycerine was used for assessment of endothelium-independent vasodilation.[1] After acquiring baseline rest images for 30 seconds, a sublingual tablet (nitroglycerine, 75 µg) was given and imaging of the artery was done continuously for 5 minutes. NID was automatically calculated as a percentage change in peak vessel diameter from the baseline. Percentage of NID [(Peak diameter - Baseline diameter)/Baseline diameter] was used for analysis. Inter- and intra-coefficients of variation for the brachial artery diameter were 1.6% and 1.4%, respectively, in our laboratory.

Reference

1. Maruhashi, T.; Soga, J.; Fujimura, N.; Idei, N.; Mikami, S.; Iwamoto, Y.; Kajikawa, M.; Matsumoto, T.; Hidaka, T.; Kihara, Y., et al. Nitroglycerine-induced vasodilation for assessment of vascular function: a comparison with flow-mediated vasodilation. *Arterioscler Thromb Vasc Biol* **2013**, *33*, 1401-1408, doi:10.1161/atvbaha.112.300934.
